# Poisonous Nature of Ozone

**Published:** 1879-02

**Authors:** 


### The Poisonous Nature of Ozone.

M. P. Thénard, in the *Comptes Rendus*, 1877, says that false views prevail among the laity and scientists concerning the action of ozone on the animal economy; for, far from being a remedy, it is one of the most energetic poisons prepared in our laboratories. Especial attention is called to the fact that, under the influence of ozone, and even when the latter is highly diluted, the blood cells contract rapidly and change their form. The pulse is retarded so markedly that, in a guinea-pig which had a normal pulse beat of 148, after remaining in an atmosphere containing but little ozone for a quarter of an hour, the pulsations sank to 130. It is possible that ozone may be a means of combating too great an increase of the temperature, but it would be very dangerous to diffuse ozone in the air of an inhab.ted room with the false hope of thereby removing the miasm. It is true that our strongest poisons may prove to be the best remedies in suitable cases; but it is first necessary to learn how to use them, in order not to be deceived as to the proper moment of their application and the dose. The author asks further: Are we indeed certain that ozone exists in the atmosphere? Its presence is recognized by the aid of a strip of paper, the color of which is more or less changed by contact with the air. But how do we know that this change is not produced by some other matter in the air, which modifies the paper in exactly the same manner as ozone? Wittmann conducted an air current through the flame of a blast lamp, and obtained an air which acted on ozonometric paper in exactly the same manner as ozone. While this air disinfected stinking water, without giving it an acid reaction, ozone did not disinfect it, and rendered it acid. It is also known that ozone at a temperature of 200° C. has no stability, while the air modified by Wittmann was exposed to a temperature which softened the glass. The question of the presence of ozone in the atmosphere, as well as of its activity, is not yet settled, and new investigations are necessary for the accurate determination of the facts.—*Half-Yearly Compendium.*

